# Bibliographical Notices

**Published:** 1839-04-20

**Authors:** 


### BIBLIOGRAPHICAL NOTICES.

*Researches on the State of the Heart, and the Use of Wine in Typhus Fever.* By William Stokes, M. D., M. R. I. A., Honorary Fellow of the King and Queen's College of Physicians. 8vo. pp. 67. Dublin Journal of Medical Science, March, 1839.

This we look upon as a valuable and practical publication, and hasten to lay an abstract of it before our readers. Dr. Stokes commences with avowing his opposition to the doctrine of localization, viewing typhus fever as an essential disease, not symptomatic of any known local lesion, an expression of opinion in which we coincide.

"There can be no doubt that the typhus of Great Britain and Ireland is a disease of the whole system, not symptomatic of any particular local lesion; showing on the one hand a tendency to a favourable termination, after a period which varies indefinitely; and on the other, being capable of destroying life *with* various lesions, or *without* any appreciable change in the solids. It is a disease on which anatomy sheds but a negative light, not telling us what it is, but rather what it is not." (p. 2.)

The typhus fever of Dublin does not appear to correspond precisely with the epidemic in Philadelphia, of 1835–6, the enteric lesions occurring there almost as constantly as in typhoid fever, or dothinenteritis, as known amongst us.

Typhus fever being a disease in which, while with a natural tendency to a spontaneous and favorable termination, the organic forces are liable to sudden and intense prostration, during which time it is necessary to support nature to enable her to combat successfully the malignant influences she is exposed to, it becomes an interesting and important question to determine how this can be best effected, and what rules, if any, exist to guide us. Old and experienced practitioners possess, frequently, an instinctive tact in the exhibition of stimulants, which they cannot communicate to their junior brethren—a knowledge gained by long, and too often a mournful experience. Dr. Stokes expresses his solemn conviction that it is to the fear of wine in typhus, as well as an ignorance of the principles which should guide its exhibition, that the immense mortality in this affection is to be mainly attributed.

"If we compare the inexperienced man with him who has had a long continued practice in fever, we may often observe that the former employs a too vigorous antiphlogistic treatment in the commencement of the disease, and delays the exhibition of stimulants until the powers of life are sunk too low, while the latter is much more cautious in husbanding the strength of his patient, and shows much less fear of resorting to wine and other stimulants. It is in determining on the use of wine in fever that the junior or inexperienced man feels the greatest difficulty; it is in its exhibition that he betrays the greatest uncertainty and fear. This is to be explained by referring to the general character of the doctrines which have prevailed within the last quarter of a century, and which are only now beginning to yield to a more rational pathology. The doctrine of an exclusive or almost exclusive solidism, which referred all diseases to visible changes of organs, which taught that inflammation was the first and principal morbid phenomenon, and that fevers were always the result of, or accompanied with, some local inflammation, was, however disguised under various denominations, the doctrine taught to the majority of our students. Their ideas were thus exclusively anatomical; inflammation formed the basis of their limited pathology, and thus instructed, they entered on the wide field of practice, most of them having never even attended a fever hospital; utterly ignorant of the nature of essential fevers, they applied, in the diseases of debility, the treatment of acute local inflammation, and delayed stimulation until nature could not be stimulated.

Let it not be supposed that in this picture I seek to make a favourable contrast between the education which I myself received, and that given to others. Far from it; I confess that it was not until several years after I commenced practice that I became fully aware of the erroneousness of what is termed the anatomical theory of disease; and I feel certain, humiliating though the confession may be, that the fear of stimulants in fever with which I was imbued, was the means of my losing many patients, whose lives would have been saved, had I trusted less to the doctrine of inflammation, and more to the lessons of experience, given to us by men who observed and wrote before the times of Bichat or of Hunter." (p. 2.)

The influence of the stimulant on the condition of the pulse, has been long admitted as the true exponent of its value, and the extensive experience of Dr. Stokes adds strong confirmation to the truth of this proposition. When the pulse becomes fuller and slower on the exhibition of stimulants, a favourable prognosis may be indulged; where its frequency is increased, or its rapidity continues, a bad result must be expected. This naturally leads one to inquire into the condition of the heart in typhus fever, and the eighteen cases by which Dr. Stokes has illustrated his paper, are so arranged, as the reader will presently see, as to exhibit, together, the state of the heart and the amount of wine used, thus affording an additional rule, drawn from the heart itself, to regulate the administration of stimulants. In typhus we have two conditions of the heart, diametrically opposite. In the one the impulse is either altogether wanting, or very feeble, with a diminished intensity of the sounds. In the other, the heart's action continues vigorous throughout the progress of the malady. Now the state of the skin is not an index of these two cardiac conditions, the surface frequently giving the sensation of intense heat, a symptom almost pathognomic of typhus, whilst the heart's action is feeble; and on the other hand, the converse holds good, an alarming rapidity in the cardiac action with every appearance of utter prostration—the patient being cold, pulseless, and livid. The condition of this organ must be determined by the application of the hand and the stethoscope to the infra-mammary and sternal regions. From the result of numerous observations, our author thinks the conclusion may be established, "*that a vigorous action of the heart in typhus, points out that stimulants will not have so beneficial an effect as in the opposite case.*"

"I now recur to the division of cases of typhus into those with and those without altered phenomena of the heart. In the first class we observe:

1. Diminution and ultimate cessation of the impulse.
2. Diminution of the intensity of the sounds.
3. Cessation of one of the sounds.

These phenomena, hitherto undescribed, are among the most interesting of those connected with the heart which I have ever observed, and I shall be able to show that they have a most important application in practice, as bearing directly on the question as to the use of stimulants in typhus fever.

I shall now present a series of cases observed particularly with reference to the heart. They are so arranged as to show first, the general symptoms; next, the phenomena of the heart and pulse; and lastly, the amount of stimulants employed.

CASE II.—*Severe Catarrhal Typhus; Failure of the Circulation; Cessation of the first Sound of the Heart; Use of Stimulants; Recovery.*

John Keefe, æt. 20, of rather muscular frame, was admitted on the 11th of April, the seventh day of fever, with severe nervous symptoms, and all the signs, both vital and physical, of an intense bronchial affection, predominating in the left lung. The skin was thickly covered with bright red petechiæ, which were confluent, forming large patches on the arms and thighs; respirations twenty-eight, laboured; pulse 120, small and very weak.

The heart's impulse was visible, and the contractions audible, but the second sound greatly predominated over the first. It was loud and distinct, while the first was very feeble, particularly at the left side of the heart.

| DATE. | GENERAL SYMPTOMS. | PHENOMENA OF CIRCULATION. | TREATMENT. |
|---|---|---|---|
| April 12. | Delirium; intense bronchial rales; insatiable thirst; diarrhœa. | Pulse 120, weaker than yesterday; impulse less perceptible; *first sound nearly inaudible;* carotid pulsations of good strength; extremities warm. | Cupping; blister to the sternum; anodyne enema, and poultices to the belly. |
| " 14. | Continual moaning; petechiæ more diffused and dark coloured. | Pulse about 112; impulse barely perceptible; over the left cavities the first sound is scarcely distinguishable, while over the right it is more so; *second sound very clear.* | Wine 10 ounces, arrow root, decoct. senegæ. |
| " 15. | Countenance improved; much delirium; bronchitis lessened; diarrhœa continues; the marks of the cupping glasses are black. | Pulse 112, contracted and compressible; no impulse of the heart under the mamma; the first sound totally inaudible, second less distinct than yesterday; on the left margin of the sternum nothing can be heard but the second sound, and this feebly. | Wine 16 oz. Blister to abdomen; anodyne enema; beef tea. |
| " 16. | Looks better; slept well; diarrhœa less. | Pulse 108, stronger and fuller; first sound audible over the whole præcordial region, second more distinct. | Repeat all. |
| " 17. | | Pulse 100; respirations 28; impulse again perceptible. | Wine 12 ounces. |
| " 18. | | Impulse still stronger, striking over a greater surface; both sounds distinctly audible at the inferior part of left side, and also to the right of the sternum; pulse 96; respirations 32. | Wine 12 ounces; jelly 2 glasses. |
| " 19. | Bronchitis much less; petechiæ fading; bowels regular. | Pulse 76, full, of good strength; heart's impulse more vigorous; sounds as yesterday. | Wine 6 ounces. |
| " 20. | | Pulse 88; phenomena of heart natural. | Wine 6 ounces. |
| " 21. | | Ditto, ditto. | Wine 4 ounces. |
| " 23. | Convalescent. Patient discharged on 2d of May, perfectly well. | | |

In this case, and that which follows, we observe the remarkable and important peculiarity of the supervention of bad symptoms of prostration and putrescence at an unusually early period of the disease. This circumstance should always excite great apprehension, and lead to the exhibition of stimulants, notwithstanding the existence of various local irritations. In both these cases the chest and abdomen were severally engaged, and in both, the early exhibition of wine not only did no harm, but was productive of the happiest effects. The existence of signs of bronchitis or enteritis in our maculated fever does not necessarily contra-indicate the free and early use of stimulants.

In examining the efficacy of wine in typhus, if we compare the case with predominance of enteric, and those with bronchial irritation, we generally find that in the latter group the stimulant is better borne, and there is a class of cases in which wine is scarcely admissible. These cases present signs of enteric irritation of great severity, alternating with violent nervous symptoms, unaccompanied by petechiæ, or other phenomena of putrescence. The use of wine is almost always injurious from its too violently exciting the brain. But in the bad petechial typhus with great prostration of strength, the existence of thirst, abdominal pain and tenderness, diarrhœa and tumefaction, should not prevent us from having recourse to wine.

I beg to draw the particular attention of my readers to the cardiac phenomena in this case; it may be right to state, that the stethoscopic observations in this and the succeeding cases were made with the greatest care.

We observed, in the first place, a progressively diminishing impulse; on the seventh day the impulse was visible at the side, but on the tenth was altogether wanting; it reappears on the twelfth, and continues to increase until the period of the patient's restoration to health.

In the second place, we find a singular modification of the sounds of the heart; the proportion between the two sounds was lost on the seventh day, the first being exceedingly feeble, the second comparatively strong; on the eighth day the first sound was scarcely audible, and on the tenth it became extinct, and we had the singular phenomenon, never before observed, of the heart in typhus fever giving but a single sound. On the eleventh day, under the influence of powerful stimulation, the first sound reappears, and the second has more vigour; on the twelfth day both sounds are distinctly audible, and on the fourteenth the phenomena of the heart are natural." (p. 8.)

The importance and truth of the following remarks must be acknowledged by every one. The state of the tongue is the great stronghold of the sympomatic p hysician.

"I greatly doubt whether there is any symptom which we can depend on as indicative of gastric inflammation in petechial typhus. That the condition of the tongue is fallacious has been established by Andral and Louis from numerous dissections, and the utility of wine and other stimulants, when the tongue is dry and brown, gives another and different description of proof. In a paper on the use of wine and opium in fever, published by my colleague, Dr. Graves, in the first volume of the Dublin Journal of Medical Science, he observes: 'In the first place, as to the tongue, *at an advanced period* of fever, I have often derived the greatest advantage from wine and opium, although the tongue was dry, the colour of old mahogany, or else coated with a yellowish brown dry fur, and protruded with difficulty, while the teeth and gums were covered with sordes; wine or porter, in moderate quantities, seem generally to agree with this tongue better than opium; in some cases, however, the latter is indispensable. For fear of misleading the reader, I must again remark that I by no means wish to assert that such a tongue uniformly, or even frequently, indicates the use of these medicines: on the contrary, this state of the tongue and mouth will often be observed at a time when leeches and antiphlogistic treatment are required. Let it be clearly understood, however, that at an advanced period of fever this state of tongue may exist, and yet wine and opium may be given boldly, provided, as I have said before, the general state of the patient seems to require it.'

Let it be recollected, that in this case we had the symptoms of a dry and brown tongue, great thirst, epigastric tenderness, and heat of skin. On the first day of treatment leeches were applied to the epigastrium, and wine exhibited to the amount of eight ounces. I have frequently leeched the epigastrium, and ordered wine on the same day, and with benefit. In our case the epigastric tenderness was lessened, but the thirst continued insatiable: the quantity of wine was doubled. Two circumstances led to this, one the extreme coldness and lividity of the extremities; and the other, the increasing indications of debility of the heart, as shown by the great indistinctness of the first sound, and the approach of the stethoscopic phenomena to what we term *the fœtal character.*

On the third day of the use of wine, and eleventh of the disease, the pulse fell from 116 to 92, and the first sound began to recover its natural intensity; this change was first *perceived over the right cavities of the heart.* This curious fact I have repeatedly observed, and I think it may be stated, that in all cases in which the first sound is lessened or obliterated, the return to the natural character is first perceived over the right side of the heart. Whatever be the cause of these interesting phenomena, it seems much more to engage the arterial than the venous side of the heart." (p. 21.)

In the secondary abdominal irritations, Dr. S. relies much on the application of large poultices, a practice for which we are indebted to Broussais, and one particularly useful, where, from early prostration, the use of leeches are forbidden. In the treatment of the same class of cases, Dr. Leco, of Dublin, employs the hip bath, and, our author avers, with success. Of the secondary affections in typhus, none is more insidious or dangerous than the bronchitis, or demands earlier or more careful attention.

Dr. Stokes "regards the action of wine upon the heart in typhus, as both sedative and stimulant;" "sedative, in diminishing its frequency; stimulant, in restoring its impulse and muscular sounds;" but when the action is favourable, these never transcend the normal limits. In no epidemic, he states, did he give so much wine as in that of last year, and in none was he ever so successful.

"Finally, I would draw the particular attention of my readers to the fact, that in the great majority of these cases the use of wine was followed by the happiest effects. I may safely refer to the cases in proof of this proposition; *I believe that in the diminished impulse, and in the feebleness or extinction of the first sound, we have a new, direct, and important indication for the use of wine in typhus fever.* In some cases, the existence of these phenomena at an early period of the disease led us to anticipate the bad symptoms, and to commence in good time the use of the great remedy; and in others, notwithstanding the existence of severe visceral irritations, the use of stimulants has been adopted with the best success, from the same indication.

It will be seen that the quantitity of wine employed in the foregoing cases was considerable. I shall exhibit in the following table the quantity given, the day on which its exhibition was commenced, and the period of the fever, as nearly as we could calculate it.

| NAME. | QUANTITY OF WINE. | DAY OF COMMENCEMENT OF WINE. | DURATION OF FEVER. |
|---|---|---|---|
| Cavanagh, - | 26 ounces. | 8th day. | 13 days. |
| Wright, - | 36 " | 14th " | 22 " |
| Devereux, | 42 " | 14th " | 16 " |
| M'Kone, - | 60 " | 12th " | 16 days; wine continued to the 20th day. |
| Wallace, - | 66 " | 11th " | 18 days. |
| Kain, - | 88 " | 9th " | 16 " |
| Smyth, - | 144 " | 10th " | 18 " |
| Edwards, - | 156 " | 10th " | 20 " |
| Quin, - | 158 " | 5th " | 14 " |
| Hickey, - | 170 " | 7th " | 17 " |

(p. 64.)

In one case the amount of stimulants employed was enormous, and the case, although of a most unpromising character, terminated happily. The patient was an elderly woman, admitted in a state of great prostration, three weeks after the invasion.

Wine, - - - 292 ounces.
Brandy, - - - 20 "
Porter, - - - 7 bottles.
Ethereal enemata, 2
Jelly, beef-tea, &c. &c.

"I may now state the conclusions to which we have arrived from our investigations of last year:

1. That the condition of the heart in typhus fever must be determined by the application of the hand and stethoscope, the pulse being an uncertain guide.

2. That a diminished impulse, or a complete absence of impulse, occurs in certain cases of typhus fever.

3. That in such cases we may observe a diminished first sound, or even an absence of the first sound.

4. That both these characters may exist with a distinct pulse.

5. That although in most cases the diminution of the impulse and first sound coexists, yet that impulse may exist without the corresponding first sound; and conversely, that the first sound may be heard, although unaccompanied by impulse.

6. That these phenomena are most evident as connected with the left side of the heart.

7. That when the impulse and first sound are lessened or lost, the return to the healthy character is observed first over the right cavities.

8. That in some cases both sounds are equally diminished.

9. That in a few cases the first sound preponderates.

10. That these phenomena indicate a debilitated state of the heart.

11. That they may occur at an early period of the disease, and thus enable us accordingly to anticipate the symptoms of general debility.

12. That the existence of these phenomena, in a case of maculated adynamic fever, may be considered as pointing out a softened state of the heart.

13. That this softening of the heart seems to be one of the secondary local lesions of typhus.

14. That the diminution or cessation of impulse, the proportionate diminution of both sounds, or the preponderance of the second sound, are direct and nearly certain indications for the use of wine in fever." (p. 66.)

---

*Boylston Prize Dissertations on*—1, *Inflammation of the Periosteum*; 2, *Eneuresis Irritata*; 3, *Cutaneous Diseases*; 4, *Cancer of the Breast. Also, Remarks on Malaria.* By Usher Parsons, M. D., late Professor of Anatomy and Surgery, Brown University, &c. &c. Boston: 1839. 8vo. pp. 248.

Zabiel Boylston, F. R. S., of Brookline, Massachusetts, introduced inoculation for small-pox into America, in 1721. The first subject was a member of his own family; and though, for a time, violently opposed by both professional and popular clamour, he ultimately saw his experiment completely triumphant. It was a descendant of this gentleman who instituted the prize which has produced the volume of essays before us. Among the successful competitors from its institution in 1803, to the present time, are many names familiar in the medical literature of the country, as Bigelow, Sewall, Caldwell, Haxall, Holmes, &c. &c. Dr. Parsons obtained the premium on four occasions,—in the years 1827, 1828, 1830, and 1835. The subjects were—1, on Inflammation of the Periosteum; 2, on Irritable Bladder; 3, on the Connexion between Cutaneous Diseases, which are not contagious, and the Internal Organs; 4, on the Diagnostic marks of Cancer of the Breast, whether it is curable. To these is appended an "Essay on the comparative influence of Malaria as a cause of Fever." This dissertation was not so fortunate as to have the premium accorded it, but received the respectful commendation of the Committee, recommending its publication and its author's name. The successful candidate on this occasion was, we believe, Prof. Caldwell, of Louisville.

These essays evidence, we think, considerable familiarity on the part of the author with his subjects, and fully warrant the distinction they received. They have appeared before in the medical journals of the day; and the favourable attention they attracted, has induced the author, we presume, to offer them in their present form.

